# CO_2_ separation using composites consisting of 1-butyl-3-methylimidazolium tetrafluoroborate/CdO/1-aminopyridinium iodide

**DOI:** 10.1038/s41598-019-53002-x

**Published:** 2019-11-12

**Authors:** Hyun Young Kim, Sang Wook Kang

**Affiliations:** 1 Department of Chemistry, Sangmyung University, Seoul, 03016 Republic of Korea; 2 Department of Chemistry and Energy Engineering, Sangmyung University, Seoul, 03016 Republic of Korea

**Keywords:** Atmospheric chemistry, Chemical engineering

## Abstract

1-Aminopyridinium iodide (iodine salt) was used in CO_2_ separation composites consisting of CdO and 1-butyl-3-methylimidazolium tetrafluoroborate (BMIM^+^BF_4_^−^). Using iodine salt, the separation performance was largely improved. The CO_2_/N_2_ selectivity was 64.6 and the permeance of CO_2_ gas was 22.6 GPU, which was about twice that of BMIM^+^BF_4_^−^/CdO composites without addition of iodine salt. These results were due to the both effect of iodine salt on the transport of the N_2_ molecules by the cyclic ring compound and the promoting transport of CO_2_ molecules by the amine groups. Moreover, the oxide layer on the surface of the CdO could enhance the CO_2_ solubility, resulting in the enhancement of separation performance. The mechanical and chemical properties were measured using SEM, Raman, TGA and FT-IR. The cross-section of coated membranes was confirmed by SEM. The coordinative interactions of iodine salts with BMIM^+^BF_4_^−^/CdO composite were observed by Raman.

## Introduction

Most of the natural gas produced worldwide includes carbon dioxide. Carbon dioxide in nature gas occupies 70% of the total volume of gas^1^.

Unfortunately, carbon dioxide could cause corrosion of pipe lines in purification process and reduce the efficiency of natural gas^1,2^. Thus, separating and removing carbon dioxide from natural gas streams and flue gases have been major concern recently, since it could prevent pipeline corrosion and reduce global warming effects^3–6^. There were conventional carbon dioxide separation technologies to have been commonly used, such as carbon capture and storage (CCS) technology, solid adsorbent, adsorption and stripping using aqueous amine as liquid^7–9^. However, these existing technologies were economically costly and have the disadvantage of operating on a large scale^10^. On the other hand, for the case of membrane technology, it complements these disadvantages since it has many advantages such as being economically efficient, small scale operation, and being environmentally friendly^11–13^.

Polymer-based membranes, especially with ether group in their chains, have contributed to strong coordination between CO and oxygen atoms of the polymer^14–17^. This strong coordination could enhance solubility of CO_2_ molecules, resulting in the enhancement of CO_2_/airy gas selectivity^18^. For example, Liu *et al*. arranged poly(ether block amide)/polysulfone (PEBA/Psf) composite hollow fiber membrane. That membrane achieved a CO_2_ permeability up to 260 Barrer (1 Barrer = 7.5 × 10^−15^*m*^3^ (STP)) m/*m*^2^s K Pa) and 40–51.5 selectivity of CO_2_/N_2_^19^. However, since poly(ethylene oxide) (PEO) has a relatively large number of ether group, its mechanical properties were relatively weak even though it has high crystallinity.

For ionic liquid (IL)-based membranes, it has various advantages such as thermally stable and had long- term stability under pressurized conditions. Using these advantages, many researches on various gas separation membranes based on ILs have been reported^20–22^. For example, membrane consisting of 1-hexyl-3-methylimidazolium nitrate (HmimNO_3_)/Cu nanoparticles (CuNPs) showed a selectivity of 6.2 for CO_2_/CH_4_ and 7.4 for CO_2_/N_2_ selectivity^23^.

In our previous study, we have studied the CO_2_ permeance and CO_2_/N_2_ selectivity by preparing membranes by adding various metal oxides to ionic liquids. For example, for the BMIMBF_4_/ZnO composite membrane, the CO_2_ permeance was 101 GPU and the CO_2_/N_2_ selectivity was 42.1^24^. The ZnO oxide layer has a strong affinity for CO_2_ and has been shown to influence the increase in solubility of CO_2_. The BMIMBF_4_/CdO composite membrane had CO_2_/N_2_ selectivity of 32.5 and a CO_2_ permeance of 57.1 GPU^25^. As a result, when the metal oxide was introduced, the oxide layer could improve the CO_2_ solubility, which has influenced the CO_2_ transport by free ions in the ionic liquid.

In this study, BMIM^+^BF_4_^−^/CdO composite membrane was used with an iodine salt containing amine group in metal oxide doped membrane. For these membranes, it was expected that the amine group in iodine salt will interact with CO_2_ molecules for solubility enhancement. Furthermore, the CdO nanoparticles have strong affinity for CO_2_ capable of enhancing the transport. It was also thought that the synergistic influence of the cyclic ring effect in iodine salt plays a role as barriers for N_2_ transport, resulting in the increase of CO_2_/N_2_ selectivity.

## Results and Discussion

### SEM images analysis

**Figure 1:**
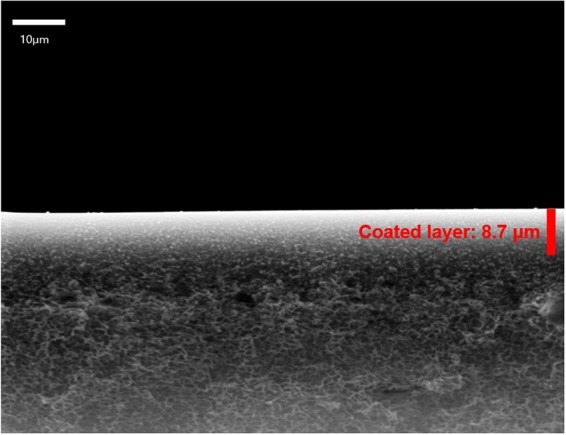
Scanning electron microscopy (SEM): BMIM^+^BF_4_^−^/CdO/iodine salt composite membrane.

As shown in Fig. 1, the morphology was examined by SEM and used to investigate the average thickness of the coating solution on the polysulfone microporous support. The average pore size of neat polysulfone was 0.1 μm and pore of surface state was also observed as microporous structure. SEM image showed that the polysulfone support was similar to the sponge like structure and the thickness of selective layer was about 8.7 μm.

Previous studies have shown that the CO_2_/N_2_ selectivity in the 1/0.007 in the composite membrane of BMIM^+^BF_4_^−^/CdO on polymer support with finger-like structure was 32.5^25^. In this study, the shape of the cross-section of the polysulfone support was sponge like structure.

### TEM images analysis

**Figure 2:**
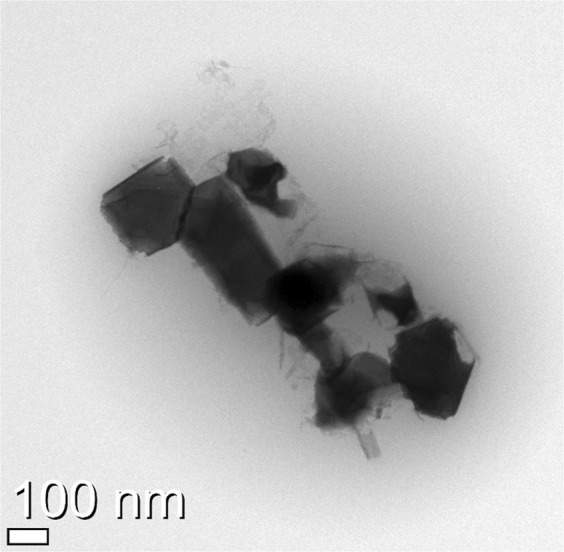
Transmission electron microscopy (TEM) image of CdO particles in BMIM^+^BF_4_^−^/CdO/iodine salt composite.

To investigate the CdO nanoparticles in BMIM^+^BF_4_^−^/CdO/iodine salt composite, TEM was observed as shown in Fig. 2. TEM image showed that the average size of generated CdO nanoparticles were ranged from 100 to 200 nm and the aggregation phenomena was observed.

### Separation performance

**Figure 3:**
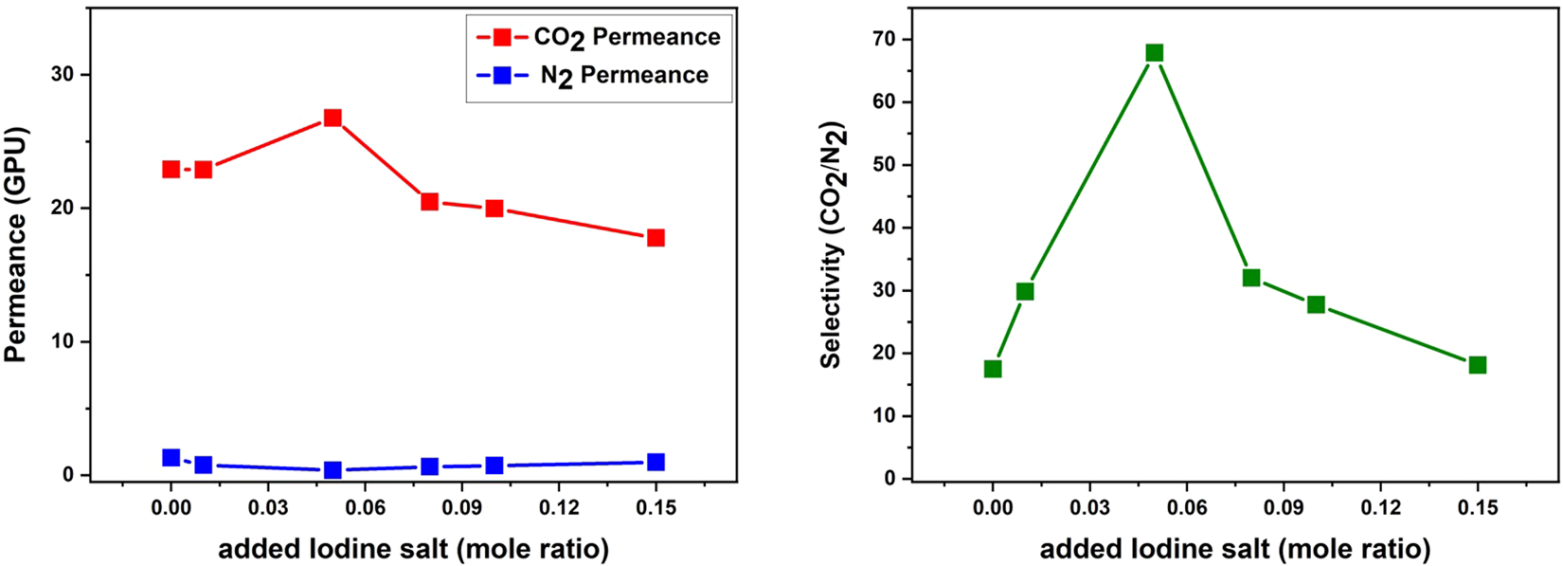
Separation performance of the BMIM^+^BF_4_^−^/CdO/iodine salt composite membranes: (**a**) single gas permeance and (**b**) CO_2_/N_2_ selectivity.

Figure 3 showed the separation of CO_2_/N_2_ using a BMIM^+^BF_4_^−^ ionic liquid containing CdO particles and iodine salt. The weight ratio of BMIM^+^BF_4_^−^/CdO was fixed at 1:0.007 and membranes with increasing mole ratio of iodine salt were tested at room temperature using a single gas (CO_2_ and N_2_). These experiments were tested three times and the single gas permeances measured for CO_2_ and N_2_ were described in Fig. 3(a). As the mole ratio of iodine salt increased, the permeance of CO_2_ increased to 0.05 mole ratio of salts and decreased above that ratio due to the aggregation phenomena of iodine in composite. These aggregation phenomena of iodine salts prevented the gas molecules from being transported through membrane, diminishing the permeance. Thus, CO_2_ permeance decreased with increasing iodine salt to 0.05 mole ratio of salts. Thus, the best separation performance was observed at 0.05 mole ratio of iodine salts as shown in Fig. 3(b).

The single gas permeance and selectivity of the composite membranes: 1/0.007 BMIM^+^BF_4_^−^/CdO and 1/0.007/0.05 BMIM^+^BF_4_^−^/CdO/iodine salt membranes were compared as shown in Table 1. Table 1 indicated that BMIM^+^BF_4_^−^/CdO composite membranes showed the CO_2_ permeance of 22.9 GPU and the selectivity of 17.6 (CO_2_/N_2_) while the CO_2_ permeance of 22.6 GPU and the selectivity of 64.6 for BMIM^+^BF_4_^−^/CdO/iodine salt composite membranes. These results were attributable to that the iodine salt acts as a hindrance to the moving of gas molecules, resulting in the decrease in overall permeance (‘barrier effect’). However, the transport of CO_2_ could be accelerated by the amine group of iodine salt. In addition, the interaction between the oxide layer formed from the dissociated CdO nanoparticles and the CO_2_ molecule also could improve the solubility of CO_2_.

**Table 1:** Single gas permeance and selectivity of the composite membranes: 1/0.007 BMIM^+^BF_4_^−^/CdO and 1/0.007/0.05 BMIM^+^BF_4_^−^/CdO/iodine salt membranes.

	CO_2_ Permeance (GPU)	N_2_ Permeance (GPU)	Selectivity (CO_2_/N_2_)
1/0.007 BMIM^+^BF_4_^−^/CdO [finger-like]	57.1	1.8	32.5
1/0.007 BMIM^+^BF_4_^−^/CdO [sponge-like]	22.9	1.3	17.6
1/0.007/0.05 BMIM^+^BF_4_^−^/CdO/iodine salt [sponge-like]	22.6	0.35	64.6

### Raman analysis

**Figure 4:**
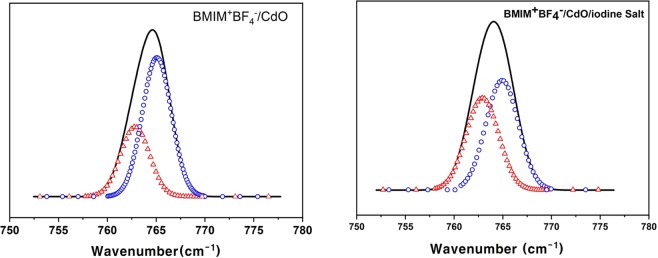
Raman spectra of (**a**) 1/0.007 BMIM^+^BF_4_^−^/CdO and (**b**) 1/0.007/0.05 BMIM^+^BF_4_^−^/CdO/iodine salt composite. Circles and triangles indicate free ion and ion pairs.

Raman was measured to investigate the interaction of iodine salt molecule in BMIM^+^BF_4_^−^/CdO composite. The Raman spectrum BMIM^+^BF_4_^−^/CdO (1/0.007) was described in Fig. 4(a), and the addition of 0.05 mol of iodine salt was shown in Fig. 4(b). The three different ionic species for various BF_4_^−^ states such as ionic aggregates, ion pairs, and free ions were observed at 777, 770, and 765 cm^−1^, respectively^26^. Table 2 compared the deconvolutions of BMIM^+^BF_4_^−^/CdO (1/0.007) and BMIM^+^BF_4_^−^/CdO/iodine salt (1/0.007/0.05) for each area of BF_4_^−^ species. In the case of BMIM^+^BF_4_^−^/CdO/iodine salt, the region of free ions was extended from 35% to 45.7%. These results suggested that new binding of BMIM ^+^ and iodine salt of BMIM^+^BF_4_^−^/CdO weakened the interaction between BMIM ^+^ and BF_4_^−^, leading to the increase in free ions of BF_4_^−^.

**Table 2:** Percentage of every ion species in the 1/0.007 BMIM^+^BF_4_^−^/CdO and 1/0.007/0.05 BMIM^+^BF_4_^−^/CdO/iodine salt composites.

	Free ions (%)	Ion pair (%)
1/0.007 BMIM^+^BF_4_^−^/CdO	35	65
1/0.007/0.05 BMIM^+^BF_4_^−^/CdO/iodine salt	45.7	54.3

### TGA analysis

**Figure 5:**
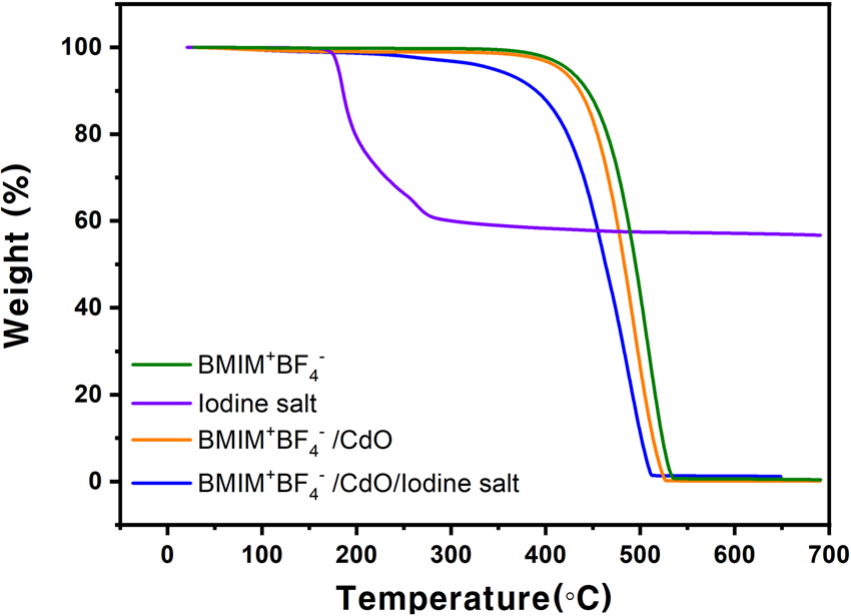
TGA graph of BMIM^+^BF_4_^−^/CdO (orange), neat BMIM^+^BF_4_^−^ (green), BMIM^+^BF_4_^−^/CdO/iodine salt (blue) and neat iodine salt (purple).

The TGA of Fig. 5 was measured at room temperature up to 600 °C. In particular, the thermal stability of the BMIM^+^BF_4_^−^/CdO composite and BMIM^+^BF_4_^−^/CdO/iodine salt composites was compared using a TGA analysis. In the case of BMIM^+^BF_4_^−^/CdO composite, the weight loss occurred at 390 ~ 530 °C while BMIM^+^BF_4_^−^/CdO/iodine salt composite lost the weight at 200 ~ 500 °C. The overall graph showed that when CdO and iodine salt were added, the stability of BMIM^+^BF_4_^−^ was gradually reduced. When CdO was added, the CdO particles were well dispersed in BMIM^+^BF_4_^−^, resulting in that the interactions between BMIM^+^BF_4_^−^ and CdO generated the decrease of thermal stability^25^. Furthermore, the addition of iodine salt caused the iodine salt to be interacted with BMIM^+^BF_4_^−^, further reducing thermal stability.

### FT-IR analysis

**Figure 6:**
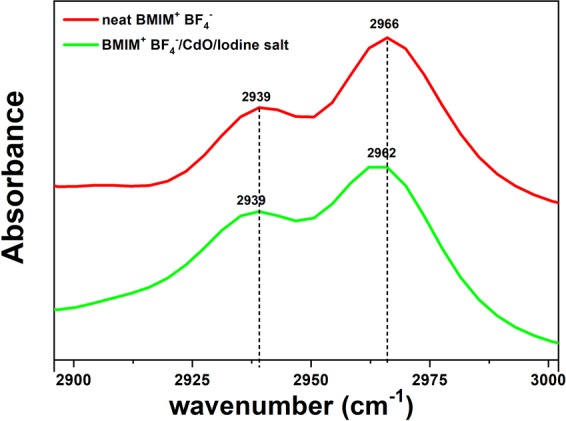
FT-IR spectra of neat BMIM^+^BF_4_^−^ and BMIM^+^BF_4_^−^/CdO/iodine salt composite.

Figure 6 showed the FT-IR spectra for the neat BMIM^+^BF_4_^−^ and BMIM^+^BF_4_^−^/CdO/iodine salt composites. The C-H stretching band the alkyl group of neat BMIM^+^BF_4_^−^ was known to be observed at 2966 cm ^−1 ^^27^. However, when iodine salt was added to neat BMIM^+^BF_4_^−^, it shifted from 2966 to 2962 cm^−1^, due to the new coordinative interaction.

**Figure 7:**
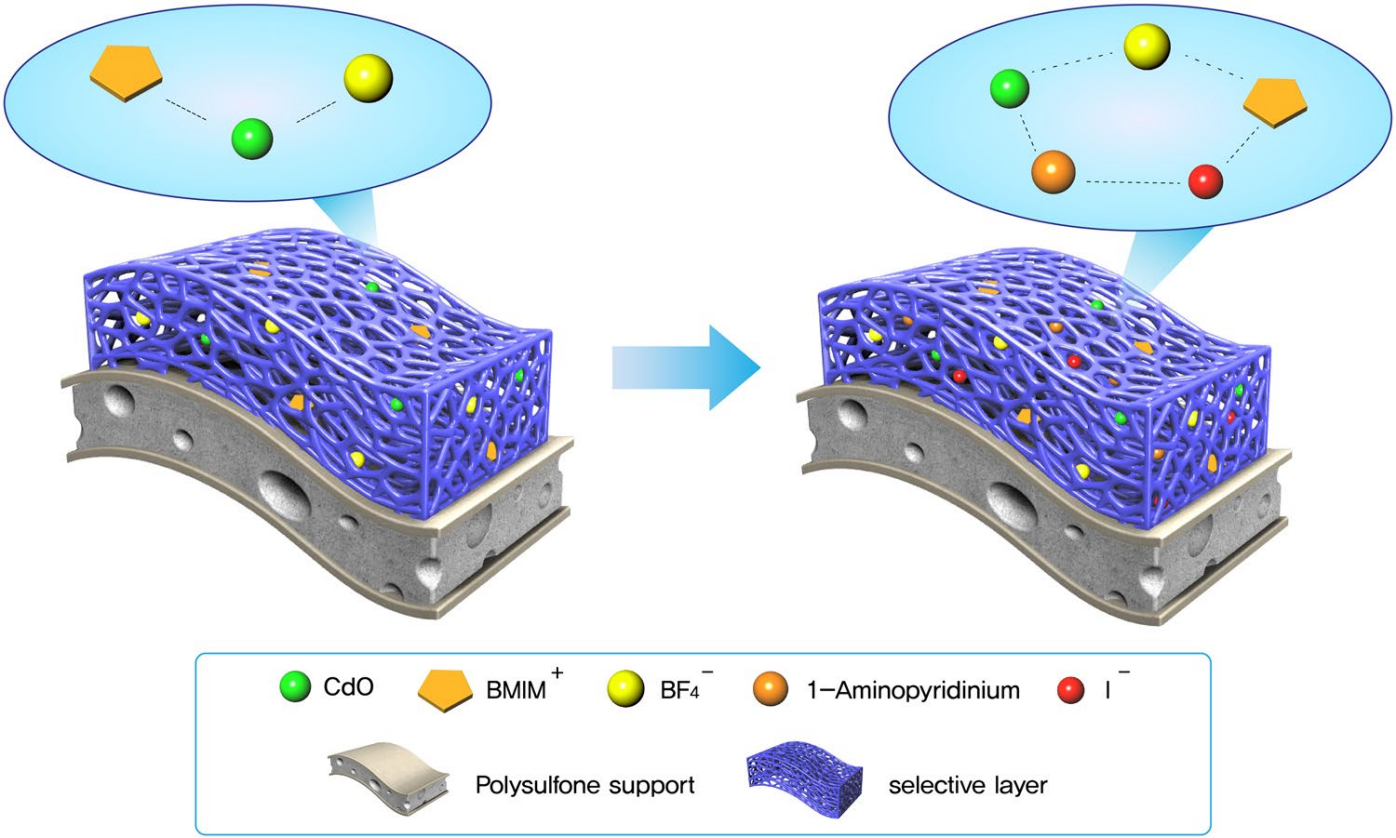
Coordination behavior in 1-butyl-3-methylimidazolium tetrafluoroborate/CdO/1-aminopyridinium iodide composite.

As shown in Fig. 7, with the new combination of BMIM^+^ and iodine salt, the existing C-H stretching band was weakened. This also indicated BF_4_^−^ became more free ions when iodine salt was added to BMIM^+^BF_4_^−^/CdO in the raman spectra.

## Conclusions

We have succeeded in preparing high selective carbon dioxide membranes consisting of BMIM^+^BF_4_^−^/CdO/iodine salt composite to facilitate the CO_2_ transport for high separation performance. The features and interactions in BMIM^+^BF_4_^−^/CdO/iodine salt composites were characterized by SEM, Raman, TGA, and FT-IR. The separation performance of BMIM^+^BF_4_^−^/CdO/iodine salt composite membrane was significantly increased compared to BMIM^+^BF_4_^−^/CdO composite. When CdO and iodine salt were incorporated into BMIM^+^BF_4_^−^, the CO_2_/N_2_ selectivity was 64.6 and the permeance of CO_2_ molecules was 22.6 GPU. These results were due to the both effect of iodine salt on the transport of the N_2_ molecules by the cyclic ring compound and the facilitating transport of CO_2_ molecules through the amine group.

## Methods

### Materials

Cadmium oxide (CdO) was supplied from Sigma-Aldrich Chemical Co. 1-Aminopyridinium iodide (Iodine salt, 97%) was supplied from Sigma-Aldrich Chemical Co. 1-Butyl-3-methyl imidazolium tetrafluoroborate (BMIM^+^BF_4_^−^) was supplied from Merck KGaA (Darmstadt, Germany). Ethyl alcohol (≥94.0%) was supplied from Daejung Chemicals & Metals. The microporous polysulfone support (average pore size = 0.1 μm) for the enhancement of mechanical property was provided by Toray Chemical Inc., Korea. All the chemicals were used without purification.

### Preparation of membranes

The membranes were prepared utilizing BMIM^+^BF_4_^−^, cadmium oxide, iodine salt and ethanol. As first step, the cadmium oxide was sonicated to be dispersed in ethanol for 5 minutes. Then, BMIM^+^BF_4_^−^ and iodine salt were added to the ethanol mixture with cadmium oxide dispersed. The solution was heated at 85 °C for 24 hours to evaporate the ethanol. Then, solution was coated onto a polysulfone microporous support and cast using a RK control coater (Model K202, Control Coater RK Print-Coat Instruments Ltd., UK). The best performance of BMIM^+^BF_4_^−^/CdO/iodine salt was observed at 1/0.007/0.05 (The ratio of BMIM^+^BF_4_^−^/CdO was described by weight ratio while BMIM^+^BF_4_^−^/iodine salt was mole ratio).

### Gas separation experiments

The all gas flow rates represented by gas permeance were determined using a bubble flow meter at the steady-state. Gas flow rates were measured with a mass flow meter at an upstream with various pressure of psig and atmospheric downstream pressure. The permeances of CO_2_ and N_2_ were measured in gas permeance units (GPU), where 1 GPU = 1 × 10^6^ cm^3^ (STP)/(cm^2^·s·cmHg) and the ideal selectivity was defined as CO_2_ permeance divided by N_2_ permeance.

### Characterization

The scanning electron microscope (SEM; JSM – 5600 LV, JEOL) characterized prepared membranes. The transmission electron microscope (TEM; Tecnai F20 G^2^–200 KV, FEI) was used to characterize the CdO nanoparticle ranges in composite. The weight loss was collected using a thermogravimetric analysis (TGA; Q50 TA Instrument) of the composite membrane in flowing N_2_. Raman spectra of for BMIM^+^BF_4_^−^/CdO solution and BMIM^+^BF_4_^−^/CdO/iodine salt solution were obtained using a BRUKER RAM II instrument at a resolution of 4 cm^−1^ at room temperature. The IR measurements were used on a VERTEX 70 FT-IR spectrometer; 16–32 scans were done at a resolution of 8 cm^−1^. A sonifier were used Branson 450 (Branson Ultrasonics Corporation, Danbury CT, USA) with a standard tip.
